# Incidence, clinical characteristics, and risk factors of acute kidney injury in patients with *Chlamydia psittaci* pneumonia

**DOI:** 10.3389/fcimb.2026.1936448

**Published:** 2026-09-18

**Authors:** Changquan Fang, Junhong Lin, Hui Huang, Liuqing Peng, Jialiu Zou, Cheng Sun, Limin Xu

**Affiliations:** 1 Department of Pulmonary and Critical Care Medicine, Huizhou Central People’s Hospital, Huizhou, Guangdong, China; 2 Department of Geriatrics, Huizhou First Hospital, Huizhou, Guangdong, China; 3 Department of Pulmonary and Critical Care Medicine, Dongkeng Hospital of Dongguan, Dongguan, Guangdong, China; 4 Department of Pulmonary and Critical Care Medicine, Guangzhou First People’s Hospital Affiliated to South China University of Technology, Guangzhou, Guangdong, China

**Keywords:** acute kidney injury, Chlamydia psittaci, pneumonia, psittacosis, risk factors

## Abstract

**Objective:**

*Chlamydia psittaci* infection can cause acute kidney injury (AKI). However, systematic studies on this issue remain limited. This study aimed to examine the incidence, clinical characteristics, and risk factors of AKI in patients with *Chlamydia psittaci* pneumonia.

**Methods:**

This study retrospectively analyzed the clinical data of patients with *Chlamydia psittaci* pneumonia who were admitted to 10 hospitals in Guangdong Province, China, between January 2019 and June 2026. Renal function parameters on admission and factors associated with AKI were also analyzed.

**Results:**

A total of 241 patients with *Chlamydia psittaci* pneumonia were included, of whom 51 developed AKI. The incidence of AKI was 21.2% and was significantly higher in patients with severe pneumonia than in those with non-severe pneumonia (*p* < 0.001). Compared with patients without AKI, the AKI group had higher proportions of males and individuals with a history of smoking, hypertension, or coronary artery disease. This group also had higher proportions of patients with dyspnea, myalgia, and altered mental status. In addition, patients with AKI had higher pneumonia severity index (PSI) scores, longer hospital stays, and higher mortality. Laboratory tests showed that C-reactive protein (CRP), CRP-to-lymphocyte ratio, procalcitonin, creatine kinase (CK), lactate dehydrogenase, aspartate transaminase, D-dimer, blood urea nitrogen, serum creatinine, N-terminal pro-brain natriuretic peptide, and high-sensitivity cardiac troponin T levels were significantly higher in the AKI group than in the non-AKI group. Logistic regression analysis showed that hypertension, a higher PSI score, and elevated CK were independent risk factors for AKI in patients with *Chlamydia psittaci* pneumonia. Among patients with AKI, Kidney Disease: Improving Global Outcomes stage 1 was the most common (74.5%), and stage 2 and stage 3 accounted for 21.6% and 3.9%, respectively. Of the 51 patients with AKI, seven died (13.7%). The remaining 44 patients (86.3%) recovered renal function after prompt anti-infective and supportive treatment.

**Conclusion:**

AKI is a serious complication of *Chlamydia psittaci* pneumonia and is associated with greater disease severity and a significantly increased risk of death. In clinical practice, particular attention should be paid to high-risk patients with hypertension, a high PSI score, and elevated CK. Early renal function monitoring and intervention should be enhanced to improve patient outcomes.

## 1 Introduction

Psittacosis is a zoonotic disease caused by *Chlamydia psittaci* (*C. psittaci*) and is primarily characterized by pulmonary involvement (Knittler and Sachse, 2015). Human infection is mainly associated with inhalation of aerosols generated from the excreta or secretions of infected birds (Zhang et al., 2022b; Wang et al., 2024). Although the incidence of psittacosis is low, *C. psittaci* is highly pathogenic and can cause not only pneumonia but also multisystem involvement (Knittler and Sachse, 2015; Wang et al., 2026). *C. psittaci* pneumonia is a distinct form of pneumonia with typical clinical manifestations including high fever, relative bradycardia, headache, myalgia, fatigue, and dyspnea. In some patients, extrapulmonary manifestations, such as liver dysfunction, splenomegaly, myocarditis, meningitis, and acute kidney injury (AKI), may also occur (Fang et al., 2026).

AKI is a common complication of pneumonia, particularly in patients with severe pneumonia (Ji et al., 2025). Previous studies have mainly focused on AKI associated with bacterial or viral pneumonia, such as COVID-19 (Marzuillo et al., 2021; Matsumoto and Prowle, 2022), whereas reports of AKI as a complication of *C. psittaci* pneumonia remain scarce and are mostly limited to case reports (Zhang et al., 2022c) or small case series (Song et al., 2026). To date, no comprehensive study has systematically evaluated the incidence, clinical characteristics, and risk factors of AKI in patients with *C. psittaci* pneumonia.

Therefore, in this study, we retrospectively analyzed a large cohort of patients with *C. psittaci* pneumonia. We assessed renal function, analyzed the clinical characteristics of patients with AKI, and explored the independent risk factors of AKI to provide evidence for early recognition and intervention in clinical practice.

## 2 Methods

### 2.1 Study design and subjects

This retrospective study included patients with *C. psittaci* pneumonia who were admitted to 10 hospitals in Guangdong Province, China, between January 2019 and June 2026: Huizhou Central People’s Hospital, Huizhou First Hospital, Huizhou Third People’s Hospital, Guangzhou First People’s Hospital, Zhongshan People’s Hospital, Dongkeng Hospital of Dongguan, Shaoguan First People’s Hospital, Boluo County People’s Hospital, Guangzhou Red Cross Hospital, and Chaozhou Central People’s Hospital. The inclusion criteria were: (1) fulfillment of the imaging and clinical diagnostic criteria for community-acquired pneumonia (Metlay et al., 2019); (2) confirmation of *C. psittaci* infection in bronchoalveolar lavage fluid or blood samples by metagenomic next generation sequencing (mNGS) or targeted next generation sequencing (tNGS); and (3) age ≥ 18 years. The exclusion criteria were: (1) chronic renal insufficiency, urinary tract obstruction, or a history of malignancy; (2) coinfection with other confirmed pathogens, such as severe acute respiratory syndrome coronavirus 2 (SARS-CoV-2), influenza virus, or *Legionella* spp.; and (3) incomplete clinical data. The patients included in this study were from multiple centers, and mNGS and tNGS testing was outsourced to different third-party laboratories. Each laboratory followed a standardized testing procedure, including sample processing, nucleic acid extraction and purification, library preparation, sequencing, and bioinformatics analysis. A pathogen detection report was subsequently issued.

### 2.2 Data collection

A standardized clinical case report form was developed to collect the following information: (1) general characteristics, including age, sex, comorbidities, smoking history, and drinking history; (2) presenting symptoms and vital signs on admission; (3) laboratory test results and chest computed tomography findings; and (4) treatment and outcomes. Pneumonia severity index (PSI) scores were calculated for all patients according to clinical parameters on admission. The PSI assigns weighted points to demographic, clinical, laboratory, and radiographic variables. The total score stratifies patients into five risk classes: Class I (0 points), Class II (1–70 points), Class III (71–90 points), Class IV (91–130 points), and Class V (>130 points), with higher classes corresponding to an increased risk of 30-day mortality (Fine et al., 1997).

### 2.3 Diagnostic criteria

AKI was diagnosed and staged as per the 2012 Kidney Disease: Improving Global Outcomes (KDIGO) criteria (Khwaja, 2012). Baseline serum creatinine (SCr) was defined as the most recent value obtained within 3 months before admission or, if unavailable, the first SCr value obtained on admission. Stages were defined as follows: Stage 1: SCr increase ≥ 26.5 μmol/L within 48h, or ≥1.5–1.9× baseline within 7 days, or urine output (UOP) <0.5 mL/kg/h for 6–12h; Stage 2: SCr increase 2.0–2.9× baseline, or UOP <0.5 mL/kg/h for ≥12h; Stage 3: SCr increase ≥3.0× baseline, or ≥353.6 μmol/L (4.0 mg/dL), or initiation of renal replacement therapy (RRT), or UOP <0.3 mL/kg/h for ≥ 24h, or anuria for ≥12h.

Severe community-acquired pneumonia was diagnosed according to the 2019 clinical practice guidelines jointly issued by the American Thoracic Society and the Infectious Diseases Society of America (Metlay et al., 2019), which include one major criterion (need for invasive mechanical ventilation or septic shock requiring vasopressors) or at least three of nine minor criteria (respiratory rate ≥ 30 breaths/minute, PaO_2_/FiO_2_ ≤250, multilobar infiltrates, confusion/disorientation, blood urea nitrogen ≥20 mg/dL, white blood cells <4,000/μL, platelets <100,000/μL, hypothermia, and hypotension requiring aggressive fluid resuscitation).

### 2.4 Statistical analysis

Statistical analyses were performed with SPSS version 25.0 (IBM Corp., Armonk, NY, USA). Normality of continuous variables was assessed using the Kolmogorov–Smirnov test. Normally distributed continuous variables are expressed as mean ± standard deviation (SD), and between-group comparisons were performed with the t test; correlation analysis was performed with Pearson correlation analysis. Non-normally distributed continuous variables are expressed as median (interquartile range). Comparisons between groups were performed using the Mann–Whitney U test, and correlation analysis was performed with Spearman rank correlation analysis. Categorical variables are expressed as number of cases (%), and comparisons between groups were performed with the χ² test or Fisher’s exact test. Variables that were statistically significant in univariate analysis, together with factors considered potentially associated with AKI based on clinical expertise, were included in multivariable logistic regression analysis. The results are expressed as odds ratios (ORs) with 95% confidence intervals (CIs). Receiver operating characteristic (ROC) curves were used to assess the predictive value of relevant indicators. Statistical significance was set at *p* < 0.05.

## 3 Results

### 3.1 Characteristics of patients with *C. psittaci* pneumonia

A total of 258 patients diagnosed with *C. psittaci* pneumonia were enrolled. Of these, five patients with chronic renal insufficiency, two with influenza virus infection, two with severe acute respiratory syndrome coronavirus 2 (SARS-CoV-2) infection, and eight with incomplete clinical data were excluded. Thus, 241 patients were included and divided into the AKI group (n = 51) and the non-AKI group (n = 190) according to whether AKI occurred (**Figure 1**).

**Figure 1:**
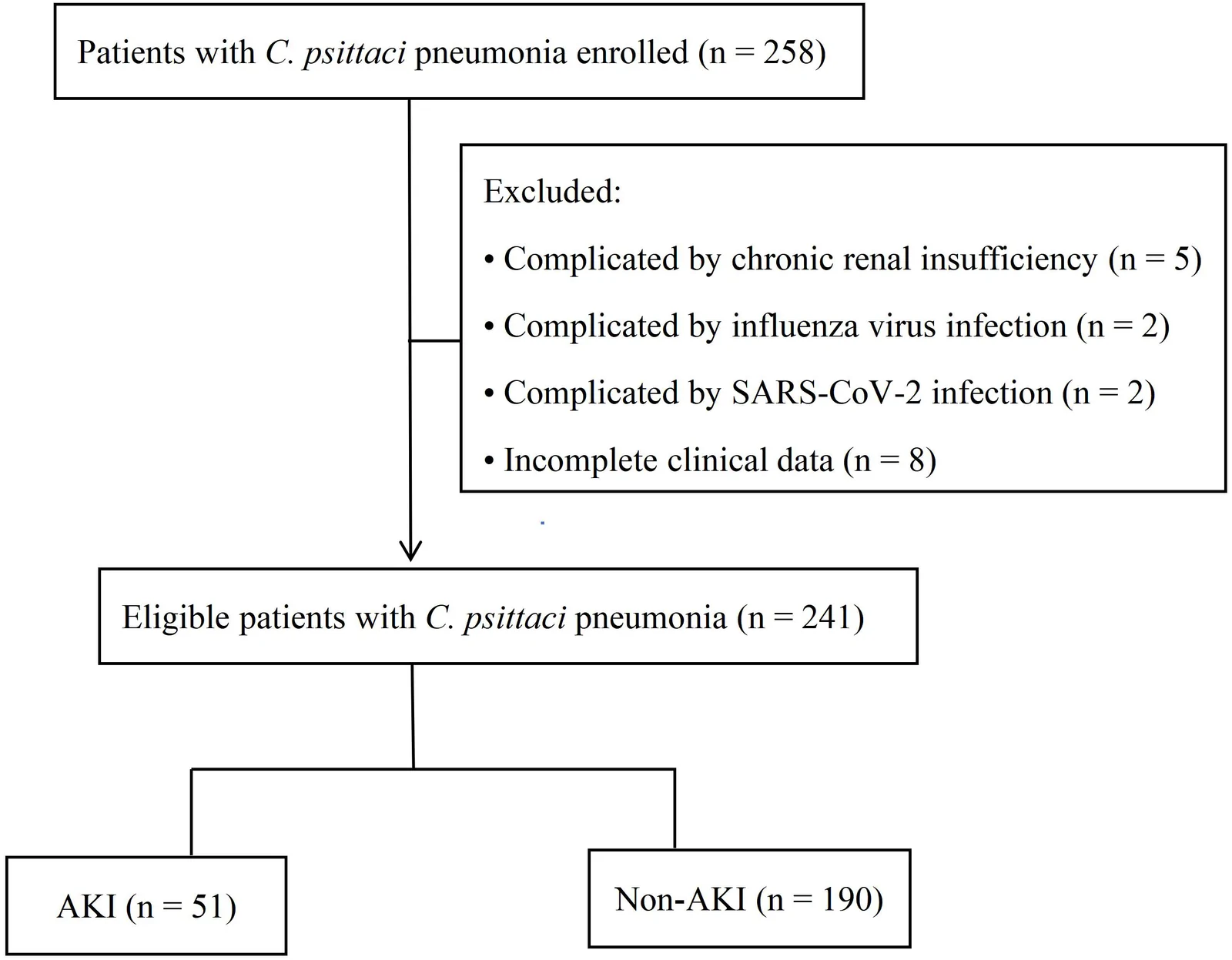
Flow-chart describing patient enrolment.

Among the 241 patients, 144 (59.8%) were male and 97 (40.2%) were female, and the mean age was 58.6 ± 12.1 years. The median duration from symptom onset to admission was 5 (4–7) days. Fifty-four patients (22.4%) had a drinking history, and 90 (37.3%) had a smoking history. A total of 119 patients (49.4%) had at least one comorbidity: hypertension in 60 (24.9%), diabetes mellitus in 48 (19.9%), and coronary artery disease in 18 (7.5%); some patients had two or more comorbidities. Compared with the non-AKI group, the AKI group had higher proportions of male patients, patients with a smoking history, and patients with hypertension or coronary artery disease (**Table 1**).

**Table 1:** Clinical features of AKI in patients with C. psittaci pneumonia.

Characteristics	AKI	Non-AKI	Total	*p* value
N	51	190	241	-
Age, years, mean ± SD	61.0 ± 12.5	58.1 ± 12.2	58.6 ± 12.1	0.261
Males, n (%)	41 (80.4)	103 (54.2)	144 (59.8)	0.001
Drinking history, n (%)	16 (31.4)	38 (20.0)	54 (22.4)	0.084
Smoking history, n (%)	27 (52.9)	63 (33.2)	90 (37.3)	0.010
BMI, kg/m^2^, median (IQR)	24.7 (21.2-26.5)	24.1 (21.2-25.4)	24.2(21.2-25.5)	0.356
Duration from symptom onset to admission, days, median (IQR)	5 (3–7)	5 (4–7)	5 (4–7)	0.127
PSI, median (IQR)	129 (94-153)	78 (59-108)	85 (63-118)	<0.001
Comorbidities, n (%)	30 (58.8)	89 (46.8)	119 (49.4)	0.129
Hypertension	21 (41.2)	39 (20.5)	60 (24.9)	0.002
Diabetes	15 (29.4)	33 (17.4)	48 (19.9)	0.056
Coronary artery disease	9 (17.6)	9 (4.7)	18 (7.5)	0.002
Initial symptoms, n (%)				
High fever	43 (84.3)	177 (93.2)	220 (91.3)	0.047
Fatigue	50 (98.0)	184 (96.8)	234 (97.1)	0.651
Headache	41 (80.4)	128 (67.4)	169 (70.1)	0.071
Chills	33 (64.7)	119 (62.6)	152 (63.1)	0.785
Dry cough	26 (51.0)	126 (66.3)	152 (63.1)	0.044
Myalgia	38 (74.5)	113 (59.5)	151 (62.7)	0.049
Dyspnea	45 (88.2)	96 (50.5)	141 (58.5)	<0.001
Altered mental status	15 (29.4)	14 (7.4)	29 (12.0)	<0.001
Gastrointestinal symptoms	4 (7.8)	22 (11.6)	26 (10.8)	0.613

AKI, acute kidney injury; BMI, body mass index; PSI, pneumonia severity index.

Among the 241 patients with *C. psittaci* pneumonia, 145 (60.2%) had proteinuria, 117 (48.5%) had hematuria, 67 (27.8%) had elevated SCr, 32 (13.3%) had elevated BUN, and 51 (21.2%) developed AKI. After further stratification by disease severity, among 103 patients with severe pneumonia, 45 (43.7%) had elevated SCr, and 40 (38.8%) developed AKI. Among the 138 patients with non-severe pneumonia, 22 (15.9%) had elevated SCr, and 11 (8.0%) developed AKI. The proportions of patients with elevated SCr or AKI were significantly higher in those with severe pneumonia than in those with non-severe pneumonia (*p* < 0.001) (**Table 2** and **Figure 2**).

**Table 2:** Analysis of laboratory test results for C. psittaci pneumonia patients with AKI.

Variables	AKI	Non-AKI	Total	*p* value
Laboratory testing, median (IQR)				
WBC, ×10^9^/L	9.0 (6.7-11.1)	8.65(6.5-10.8)	8.8 (6.5-10.9)	0.344
Neutrophil Count, ×10^9^/L	7.8 (5.5-10.7)	7.0 (4.9-9.4)	7.2 (5.1-9.4)	0.087
Lymphocyte Count, ×10^9^/L	0.5 (0.4-0.9)	0.7 (0.5-1.1)	0.7 (0.5-1.1)	0.003
Hemoglobin, g/L	121 (107-133)	123 (110-134)	122 (109-134)	0.806
Platelet count, ×10^9^/L	169 (131-207)	204 (152-256)	194 (149-244)	0.001
CRP, mg/L	226 (170-290)	161 (99-212)	177 (110-239)	<0.001
CLR	444 (234-675)	204 (100-365)	240 (109-442)	<0.001
Albumin, g/L	32.3 (27.0-35.4)	33.5 (28.7-36.9)	33.1 (28.4-36.4)	0.117
PCT, ng/ml	4.0 (0.9-16.1)	0.4 (0.1-0.9)	0.5 (0.2-1.7)	<0.001
CK, U/L	661 (239-4752)	171 (79-436)	229 (91-737)	<0.001
LDH, U/L	424 (293-730)	302 (235-452)	331 (248-516)	<0.001
ALT, U/L	59 (37-107)	57 (33-83)	57 (34-90)	0.316
AST, U/L	111 (55-250)	65 (36-112)	70 (40-129)	0.001
D–dimer, ng/mL	3190 (2130-8210)	1590 (1020-3235)	2050 (1080-4023)	<0.001
BUN, mmol/L	8.1 (6.5-10.8)	4.5 (3.5-6.2)	5.2 (3.7-7.1)	<0.001
SCr, umol/L	123 (114-148)	72 (61-85)	81 (63-97)	<0.001
NT-proBNP, pg/mL	572 (153-1455)	219 (112-525)	284 (125-726)	0.002
hs-cTnT, ng/L	20.9 (11.7-36.1)	10.0 (6.0-15.0)	11.0 (7.0-21.1)	<0.001
Sodium, mmol/L	133 (129-137)	135 (131-137)	135 (131-137)	0.106
Potassium, mmol/L	3.5 (3.2-3.9)	3.5 (3.2-3.8)	3.5 (3.2-3.9)	0.786
Elevated BUN, n (%)	23 (45.1)	9 (4.7)	32 (13.3)	<0.001
Proteinuria, n (%)	44 (86.3)	101 (53.2)	145 (60.2)	<0.001
Proteinuria (+)	25 (49.0)	67 (35.3)	92 (38.2)	0.049
Proteinuria (++)	13 (25.5)	27 (14.2)	40 (16.6)	0.043
Proteinuria (+++)	6 (11.8)	7 (3.7)	13 (5.4)	0.020
Hematuria, n (%)	35 (68.6)	82 (43.2)	117 (48.5)	0.001
Hematuria (+)	12 (23.5)	51 (26.8)	63 (26.1)	0.710
Hematuria (++)	8 (15.7)	15 (7.9)	23 (9.5)	0.102
Hematuria (+++)	15 (29.4)	15 (7.9)	30 (12.4)	<0.001

ALT, alanine transaminase; AST, aspartate transaminase; AKI, acute kidney injury; BUN, blood urea nitrogen; CLR, C-reactive protein to lymphocyte ratio; CK, creatine kinase; CRP, C-reactive protein; hs-cTnT, high-sensitivity cardiac troponin T; LDH, lactate dehydrogenase; NT-proBNP, N-terminal fragment brain natriuretic peptide; PCT, procalcitonin; SCr, serum creatinine; WBC, white blood cells.

**Figure 2:**
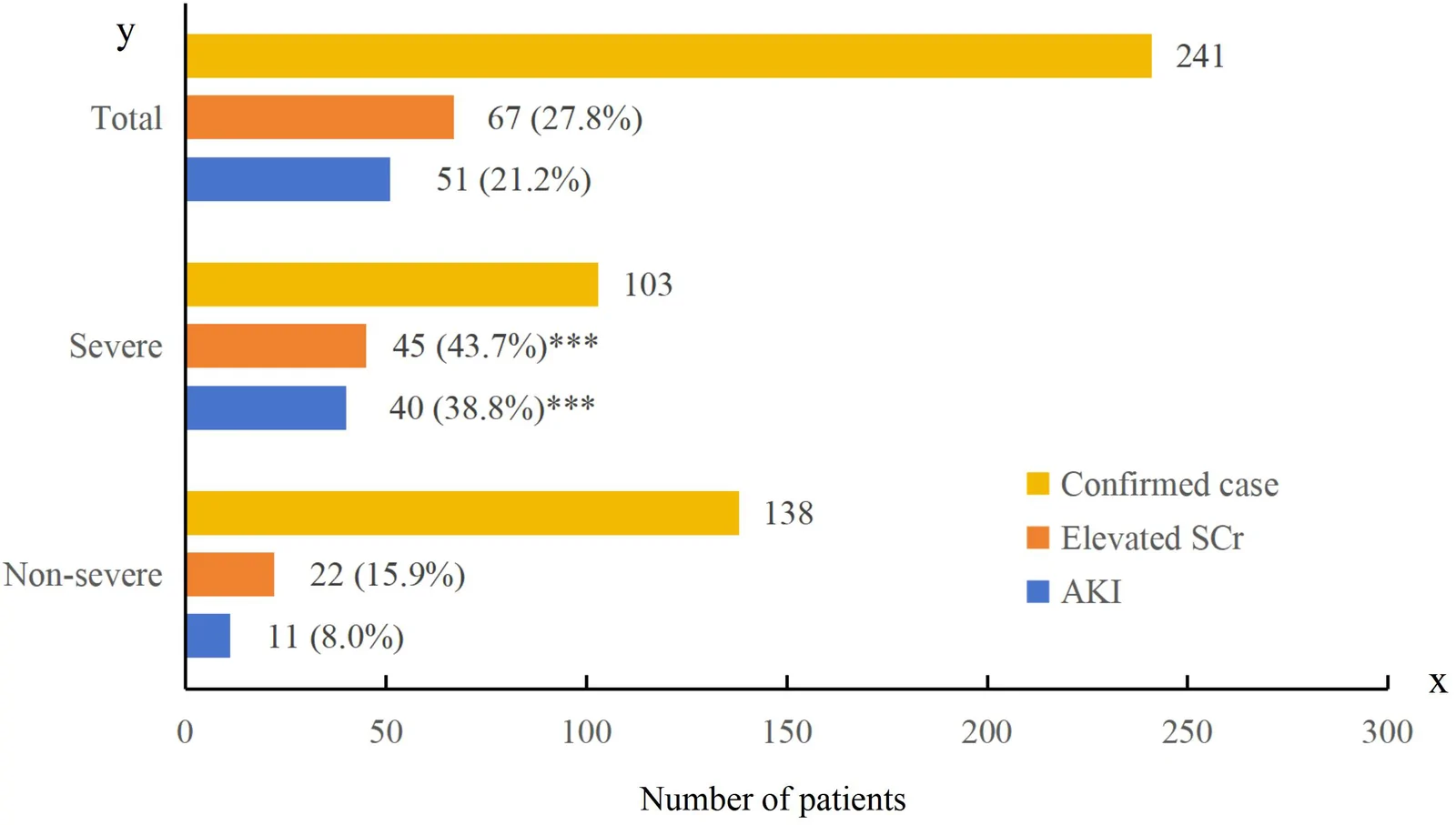
Renal function abnormality on admission in patients with C. psittaci pneumonia by disease severity. Data are presented as number (percentage). Comparisons between groups were performed using the χ² test. *P < 0.05, **P < 0.01, and ***P < 0.001 compared with non-severe pneumonia. (Bars represent number of patients).

Among the 51 patients with AKI, 38 (74.5%) had KDIGO stage 1 AKI, 11 (21.6%) had stage 2, and 2 (3.9%) had stage 3. Among the 51 patients with AKI, five patients (9.8%) received continuous renal replacement therapy, and seven patients (13.7%) died.

### 3.2 Clinical characteristics of AKI in patients with *C. psittaci* pneumonia

All patients presented with fever, and 91.3% had high fever. Other common symptoms included fatigue, headache, chills, dry cough, myalgia, and dyspnea. Some patients also developed altered mental status (confusion/disorientation) and gastrointestinal symptoms. Compared with the non-AKI group, the AKI group had higher proportions of patients with dyspnea, myalgia, and altered mental status, as well as a higher proportion of patients requiring invasive mechanical ventilation. The AKI group also had higher PSI scores, longer hospital stays, and higher mortality. Compared with the non-AKI group, patients in the AKI group had significantly higher C-reactive protein (CRP), CRP-to-lymphocyte ratio (CLR), procalcitonin (PCT), creatine kinase (CK), lactate dehydrogenase (LDH), aspartate transaminase (AST), D-dimer, blood urea nitrogen (BUN), SCr, N-terminal pro-brain natriuretic peptide (NT-proBNP), and high-sensitivity cardiac troponin T levels (hs-cTnT) and significantly lower lymphocyte and platelet counts (*p* < 0.05; **Tables 1**–**3**).

**Table 3:** Treatment and prognosis for C. psittaci pneumonia patients with AKI.

Characteristics	AKI	Non-AKI	Total	*p* value
N	51	190	241	-
Treatments				
Tetracycline, n (%)	20 (39.2)	64 (33.7)	84 (34.9)	0.462
Quinolones, n (%)	13 (25.5)	70 (36.8)	83 (34.4)	0.130
Azithromycin, n (%)	1 (2.0)	0 (0)	1 (0.4)	0.212
Combination therapy with target drugs, n (%)	17 (33.3)	56 (29.5)	73 (30.3)	0.594
Glucocorticoid therapy, n (%)	4 (7.8)	8 (4.2)	12 (5.0)	0.287
Anticoagulant therapy, n (%)	5 (9.8)	16 (8.4)	21 (8.7)	0.756
HFNC, n (%)	13 (25.5)	30 (15.8)	43 (17.8)	0.108
Non-invasive ventilation, n (%)	1 (2.0)	5 (2.6)	6 (2.5)	1.0
Invasive mechanical ventilation, n (%)	22 (43.1)	23 (12.1)	45 (18.7)	<0.001
ECMO, n (%)	3 (5.9)	5 (2.6)	8 (3.3)	0.371
Continuous renal replacement therapy, n (%)	5 (9.8)	0 (0)	5 (2.1)	<0.001
Outcomes				
Death, n (%)	7 (13.7)	2 (1.1)	9 (3.7)	<0.001
Total hospital stay, days, median (IQR)	13 (10–23)	10 (7–12)	10 (8–13)	<0.001

AKI, acute kidney injury; ECMO, extracorporeal membrane oxygenation; HFNC, high-flow nasal cannula oxygen therapy.

The correlation analysis showed that BUN and SCr were significantly positively correlated with PSI score, CLR, PCT, AST, CK, LDH, D-dimer, NT-proBNP, and hs-cTnT but negatively correlated with platelet count (**Table 4**).

**Table 4:** Correlation analysis of SCr and BUN with other indicators.

Index	BUN		SCr	
	r value	p value	r value	p value
PSI	0.594	<0.001	0.377	<0.001
WBC	0.198	0.002	0.085	0.189
PLT	-0.180	0.005	-0.173	0.007
CLR	0.357	<0.001	0.288	<0.001
PCT	0.548	<0.001	0.444	<0.001
ALT	0.122	0.058	0.062	0.377
AST	0.295	<0.001	0.157	0.015
CK	0.248	<0.001	0.333	<0.001
LDH	0.343	<0.001	0.171	0.009
D-dimer	0.389	<0.001	0.214	0.001
NT-proBNP	0.233	0.001	0.146	0.047
hs-cTnT	0.427	<0.001	0.300	<0.001

ALT, alanine transaminase; AST, aspartate transaminase; BUN, blood urea nitrogen; CLR, C-reactive protein to lymphocyte ratio; CK, creatine kinase; hs-cTnT, high-sensitivity cardiac troponin T; LDH, lactate dehydrogenase; NT-proBNP, N-terminal fragment brain natriuretic peptide; PCT, procalcitonin; PLT, platelet; PSI, pneumonia severity index; SCr, serum creatinine; WBC, white blood cells.

### 3.3 Risk factors for AKI in patients with *C. psittaci* pneumonia

Factors associated with AKI were selected for inclusion in the multivariable logistic regression analysis based on the literature review, the number of AKI events, and the results of multicollinearity testing. The analysis was then performed to assess the independent associations of these factors with AKI. With AKI as the dependent variable and male sex, hypertension, PSI score, CLR, and CK as independent variables, the logistic regression analysis showed that hypertension, a higher PSI score, and elevated CK were independent risk factors for AKI in patients with *C. psittaci* pneumonia (**Table 5**).

**Table 5:** Risk factors for AKI in multivariate logistic regression model.

Risk factors	*p* value	*OR*	95% CI
Male	0.239	1.698	0.703-4.096
Hypertension	0.021	2.518	1.148-5.527
PSI	0.000	1.126	1.035-1.322
CLR	0.620	1.029	0.918-1.154
CK	0.036	1.247	1.161-1.562

AKI, acute kidney injury; CLR, C-reactive protein to lymphocyte ratio; CI, confidence interval; CK, creatine kinase; OR, odds ratio; PSI, pneumonia severity index.

ROC curve analysis was used to assess the predictive value of PSI score and CK for AKI in patients with *C. psittaci* pneumonia (**Table 6** and **Figure 3**). The area under the curve for PSI score and CK were both greater than 0.7 (both *p* < 0.001). These findings indicate good predictive performance. At a PSI score of 112, the sensitivity and specificity for predicting AKI were 71.4% and 76.0%, respectively. At a CK level of 411 U/L, the corresponding sensitivity and specificity were 65.3% and 74.3%, respectively.

**Table 6:** The predictive efficacy of PSI score and CK for AKI.

Index	AUC (95% CI)	Optimal cutoff	Sensitivity(%)	Specificity(%)	*p* value
PSI	0.778 (0.712-0.865)	112	71.4	76.0	<0.001
CK	0.776 (0.652-0.819)	411 U/L	65.3	74.3	<0.001

AUC, area under the curve; CI, confidence interval; CK, creatine kinase; PSI, Pneumonia Severity Index.

**Figure 3:**
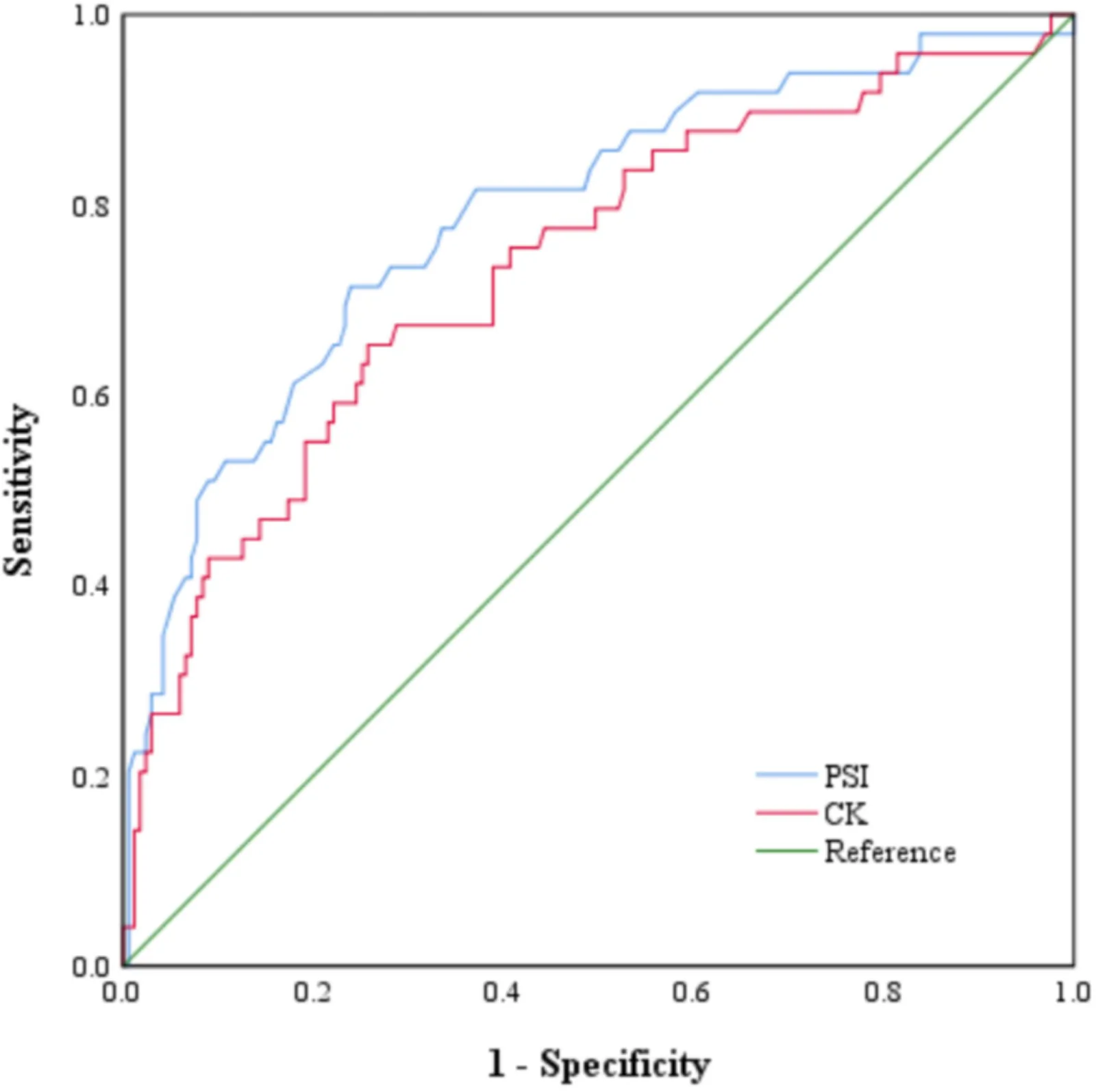
ROC curve of PSI and CK in predicting *C. psittaci* pneumonia complicated with AKI.

## 4 Discussion

This study evaluated the incidence, clinical characteristics, and risk factors associated with AKI in a relatively large cohort of patients with *C. psittaci* pneumonia. The results showed that the incidence of AKI was 21.2%. This incidence was significantly higher than that reported in patients with typical community-acquired pneumonia (Song et al., 2026; Akram et al., 2010). A study by Song et al. (2026) also reported an AKI incidence of 34.0% in patients with *C. psittaci* pneumonia. In addition, we found that 60.2% of patients had proteinuria and 48.5% had hematuria. These findings suggest that although *C. psittaci* pneumonia is caused by a relatively uncommon pathogen, the possibility of renal involvement should not be overlooked.

The mechanisms underlying kidney injury associated with *C. psittaci* infection remain unclear. Based on the literature and the clinical characteristics of patients with AKI in this study, several potential mechanisms may contribute to renal injury. First, direct pathogen invasion: *C. psittaci* is an obligate intracellular bacterium with broad host cell tropism and can infect a wide range of host cells, from epithelial cells to macrophages. After infection, it may spread hematogenously and directly invade renal tissue (Knittler and Sachse, 2015; Xu et al., 2025). Second, infection may trigger a systemic inflammatory response. *C. psittaci* is highly pathogenic and can induce an excessive inflammatory response after infection (Zhang et al., 2022a), leading to renal microcirculatory dysfunction and ischemic tubular injury (Hato and Dagher, 2025). Third, multiple organ dysfunction may result in secondary kidney injury. *C. psittaci* infection often causes multiple organ dysfunction syndrome (Wang et al., 2024; Huang et al., 2026), and complications such as respiratory failure, heart failure, and hepatic insufficiency may further aggravate renal ischemia, hypoxia, and metabolic disturbances, thereby contributing to kidney injury (Komaru et al., 2024; Che et al., 2026).

In this study, the incidence of AKI was significantly higher in patients with severe pneumonia than in those with non-severe pneumonia, and a high PSI score was identified as an independent risk factor. These findings indicate that the development of AKI is closely associated with overall disease severity. In addition, logistic regression analysis showed that hypertension and elevated CK were also independent risk factors. Patients with hypertension often have microvascular dysfunction and reduced renal vascular reserve (Zhang et al., 2023; Durante et al., 2024), making them more susceptible to decompensation under the stress of infection and to developing prerenal and ischemic kidney injury. A large cohort study showed that hypertension was an independent predictor of COVID-19-associated AKI (Jewell et al., 2021). Notably, elevated CK warrants particular attention as an independent risk factor. This finding not only reflects the extent of systemic myositis caused by infection but also suggests that some patients may have concomitant rhabdomyolysis. Myoglobin cast formation in the renal tubules and direct cytotoxic effects may jointly contribute to the development and progression of AKI (Młynarska et al., 2022; Xu et al., 2026).

This study showed that patients in the AKI group had more severe systemic manifestations, including high fever, myalgia, and altered mental status, and laboratory findings also indicated multisystem involvement. CRP, PCT, D-dimer, NT-proBNP, and hs-cTnT levels were all significantly elevated in this group. This suggests that inflammatory activation, coagulation dysfunction, myocardial injury, and kidney injury may interact with one another in a complex network (Che et al., 2026; Herrlich, 2022). Notably, the markedly elevated D-dimer in the AKI group suggests the presence of overt intravascular coagulation, which may serve as a common pathogenic driver that perpetuates concurrent myocardial, hepatic, and cerebral dysfunction. This pattern is consistent with the definition of MODS, and the cumulative number of failing organs, rather than AKI per se, likely determines the ultimate prognosis.

In this study, most patients with AKI had KDIGO stage 1 AKI, and renal function recovered in 86.3% of survivors. These findings suggest that the majority of cases were classified as KDIGO stage 1, and that renal dysfunction was reversible in the majority of survivors. In clinical practice, renal function should therefore be monitored closely in high-risk patients, particularly those with hypertension, a high PSI score, and elevated CK. Timely targeted antimicrobial therapy, including tetracyclines, macrolides, and fluoroquinolones (Balsamo et al., 2017; Garin et al., 2022), and renal supportive therapy may contribute to favorable long-term outcomes in most patients. However, the mortality rate in the AKI group was 13.7% and was significantly higher than that in the non-AKI group. Thus, the development of AKI may serve as a marker of more severe disease and poorer prognosis.

This study has several limitations. First, its retrospective design is subject to confounding bias, and the causal relationship requires confirmation in prospective studies. Second, only admission data and short-term outcomes were included, and the lack of long-term follow-up precluded assessment of the long-term impact of AKI on the development or progression of chronic kidney disease. Third, no renal biopsy was performed; therefore, the underlying renal injury results from direct pathogen invasion or indirect inflammatory damage remains uncertain and warrants further investigation. In addition, we did not measure novel urinary biomarkers of tubular injury, such as neutrophil gelatinase-associated lipocalin (NGAL) and kidney injury molecule-1 (KIM-1).

## 5 Conclusion

The high incidence and severity of AKI associated with *C. psittaci* pneumonia should be fully recognized in clinical practice. For high-risk patients with hypertension, a high PSI score on admission and markedly elevated serum CK, proactive fluid management, avoidance of nephrotoxic medications, and dynamic monitoring of renal function should be implemented to improve overall patient outcomes.

## Data Availability

The raw data supporting the conclusions of this article will be made available by the authors, without undue reservation.
